# Impact of tissue adhesives on the prevention of anastomotic leakage of colonic anastomoses: an in vivo study

**DOI:** 10.1007/s00384-017-2834-4

**Published:** 2017-05-23

**Authors:** Konstantinos A. Vakalopoulos, Joanna W. A. M. Bosmans, Kevin W. Y. van Barneveld, Ruben R. M. Vogels, Geesien S. A. Boersema, Zhouqiao Wu, Marion J. J. Gijbels, Johannes Jeekel, Gert-jan Kleinrensink, Nicole D. Bouvy, Johan F. Lange

**Affiliations:** 1 Department of Surgery, Erasmus University Medical Center Rotterdam, Rotterdam, The Netherlands; 2 Department of Surgery, Research Institute NUTRIM, Maastricht University Medical Center, Maastricht, The Netherlands; 3 Department of Pathology and Molecular Genetics, Research Institute CARIM, Maastricht University Medical Center, Maastricht, The Netherlands; 4 Department of Medical Biochemistry, Academic Medical Center, University of Amsterdam, Amsterdam, The Netherlands; 5 Ward I of Gastrointestinal Cancer Center, Key Laboratory of Carcinogenesis and Translational Research (Ministry of Education), Peking University Cancer Hospital & Institute, Beijing, 100142 China; 6 Erasmus University Medical Center, Room Ee-173: Laboratory of Experimental Surgery, Postbus 2040, 3000 CA Rotterdam, The Netherlands

**Keywords:** Anastomosis, Colorectal surgery, Anastomotic leakage, Tissue adhesives

## Abstract

**Background:**

Tissue adhesives (TA) may be useful to strengthen colorectal anastomoses, thereby preventing anastomotic leakage (AL). Previous studies have identified cyanoacrylate (CA) TAs as the most promising colonic anastomotic sealants. This study investigates the protective effects of sealing colonic anastomoses with various CAs.

**Materials and methods:**

Fifty-five Wistar rats underwent laparotomy and transection of the proximal colon. An anastomosis was created with 4 interrupted sutures followed by either application of Histoacryl Flexible, Omnex, Glubran 2, or no TA seal. An additional control group was included with a 12-suture anastomosis and no TA seal. After 7 days, the rats were sacrificed and scored for the presence of AL as the main outcome. Secondary outcomes were the occurrence of bowel obstruction, adhesions, and anastomotic bursting pressure. Histological evaluation was performed.

**Results:**

The highest AL rate was found in the Glubran 2 group (7/11), followed by the 4-sutures group without TA (5/11), and the Omnex group (5/11). Histoacryl Flexible showed the lowest AL rate (2/11). In the control group, only one rat showed signs of AL. Histologically, the highest influx of inflammatory cells was found in the 4-suture group without TA and for Omnex and Glubran 2. Histoacryl Flexible caused more mature collagen deposition when compared to the other TA groups.

**Conclusions:**

Histoacryl Flexible showed the lowest leakage rate compared to the other TA groups and to the 4-suture control group. Glubran 2 showed the highest AL rate and a high inflammatory response. Histoacryl Flexible was associated with the presence of more mature collagen and seems to promote anastomotic healing.

## Introduction

Recently, the idea of sealing an anastomosis externally with a tissue adhesive is gaining popularity and has been linked to promising results [1]. The benefit of such a technique is that a surgeon can create an anastomosis in a conventional manner using sutures or staples and perform an intra-operative anastomotic patency test, before applying an extra layer of protection on the serosal surface of the anastomosed colon. Of the various available tissue adhesives (TAs), a special interest has arisen for cyanoacrylate (CA) TAs [2]. CA is a type of chemical polymer, also known as “superglue.” Several experimental studies have been performed using CA glues to prevent AL, yielding ambiguous results [1, 2]. This may be partly due to a large spectrum of experimental methodology in the various studies, in which large differences exist in the used animal models, TA dosage, and experimental end points. This is a well-recognized problem in the field of experimental research on colorectal anastomoses [3]. In the current study, we use a rat model to simulate high rates of AL based on the creation of a mechanically insufficient colonic anastomosis, sealed by a protective barrier of one of three different CA glues. The aim of this study is to identify promising CAs for the prevention of AL, which may prevent the intraperitoneal leakage of bowel contents.

## Methods

### Study design

Three clinically available CAs were included in this study: Histoacryl Flexible (B. Braun, D; n-butyl-2-CA and a softener), Omnex (Ethicon, USA; 2-octyl/butyl lactoyl CA), Glubran 2 (GEM S.r.l., IT; n-butyl-2 CA and methacryloxy sulfolane), or no TA seal. A positive, 12-suture, control group as well as a negative, 4-suture control group were included, which were not sealed by a TA. Rat allocation was performed in a randomized manner using a lottery system. Follow-up was 7 days.

### Animals

Male specified-pathogen-free Wistar rats (250–350 g) were housed at the Central Animal Facility of the Maastricht University Medical Center, the Netherlands. The experimental protocol complied with the Dutch Animal Experimentation Act and was approved by the local Animal Experimental Committee.

### Surgical procedure

A 5-cm midline incision was made; the cecum was identified and exteriorized onto moist sterile gauzes. The ascending colon was transected 2 cm distally to the cecum, without damaging the mesenteric vessels. An insufficient end-to-end colo-colonic anastomosis was created using four evenly distributed polypropylene 6/0 sutures (Prolene; Ethicon, USA). After construction of the anastomosis, in the TA groups, 0.025 mL of TA was applied evenly to the anastomotic site using the provided applicators. Care was taken to avoid spillage into the abdomen and, if necessary, a blunt needle was used to accurately guide the TA around the anastomosis. Curing time varied based on the manufacturer’s guidelines. In the control group, 12 sutures were used instead of 4, obtaining a sufficient anastomosis.

### Outcome measures

The main outcome of the study was anastomotic leakage (AL), including macroscopic anastomotic dehiscence, fecal peritonitis, or large anastomotic abscesses. Upon sacrifice, signs of leakage or TA-related complications were noted, that is, the presence of intraperitoneal abscesses or fecal matter and mechanical ileus. Abscess formation was scored using the following scoring method: (1) one or several peri-anastomotic millimetric abscesses, (2) abscess covering up to ¼ of anastomotic circumference, (3) Large abscess; more than 1/4 of anastomotic circumference, and (4) intra-abdominal abscess formation. AL was defined as the presence of fecal peritonitis or an abscess score of >2 [4, 5]. The Zühlke score, which depicts the tenacity of intra-abdominal adhesions, was also determined [6]. Anastomotic bursting pressure testing was carried out during which the maximum bursting pressure was recorded for each rat.

### Histological evaluation

Standard hematoxylin-eosin (H&E) staining was performed. Specimens were scored based on inflammation, fibroblast activity, collagen deposition, and neoangiogenesis according to the Ehrlich and Hunt numerical scale as modified by Phillips et al., in which 0: no evidence, 1: occasional evidence, 2: light scattering, 3: abundant evidence, and 4: confluent cells or fibers [7].

### Evaluation of collagen formation

Tissue sections were stained for collagen using Picro Sirius red, as previously described [8]*.* It was chosen not to include collagen staining in the 12-suture control group due to ethical reasons, as these findings are well-known and have been reported in numerous recent studies [9–11]. Collagen percentage of anastomotic tissue was calculated. Maturity level of collagen was estimated by calculating the red (mature fibers, collagen type I) versus green (immature fibers, collagen type III) area ratio using the Qwin morphometry-system (Leica QWin V3.5.1, Leica Microsystems).

### Statistics

One-way ANOVA was used in case of continuous variables, with a Bonferroni post-hoc test. A *χ* ^2^ test or Fisher’s exact was used in case of categorical variables. A *p* value ≤0.05 was considered statistically significant. All analyses were performed using IBM SPSS Statistics, version 21.0 for Mac (IBM SPSS, USA).

## Results

### Anastomotic leakage

Both in the 4-suture non-TA group and in the Glubran 2 group, one rat died prior to completion of the follow-up period due to fecal peritonitis caused by AL. Except for these two rats, AL only consisted of the presence of anastomotic abscesses. In the 12-suture control group, AL occurred in one rat, associated with an abscess score of 1. In the 4-suture non-TA group, four rats showed signs of AL in the form of abscess formation, with an abscess score of 2 in 3 rats and an abscess score of 4 in 1 rat. In the TA groups, there was a large difference in AL-rate. Glubran 2 had the highest AL rate, consisting of 1 total anastomotic dehiscence and subsequent fecal peritonitis, and 6 cases of abscess formation. Abscess scores in this TA group ranged from 1 to 4.5 rats in the Omnex group and 2 rats in the Histoacryl Flexible group showed signs of AL in the form of abscess formation, with maximum abscess scores of 4 and 2, respectively. Statistical analysis shows that Glubran 2 had a significantly higher amount of abscesses when compared to the positive control group (*p* = 0.013) and Histoacryl Flexible (*p* = 0.049).

### Clinical outcomes

The negative control group, in which no TA was used, showed the highest rate of weight loss. For the TA groups, the Glubran 2 group showed the most weight loss on postoperative day 7, significantly higher than in the control group (*p* < 0.01) and the Histoacryl Flexible group (*p* < 0.01). Mechanical ileus rate varied significantly between the TA groups, with Glubran 2 showing the highest ileus rate, significantly higher than the negative control group (*p* = 0.01). The number and Zühlke score of adhesions did not differ significantly between the experimental groups.

### Anastomotic bursting pressure

The highest anastomotic bursting pressure (ABP) was found in the 12-suture control group (272 mmHg ±70) and differed significantly from the 4-suture no TA group (147 mmHg ±37, *p* < 0.01). The use of TA resulted in an increase in ABP in all 3 TA groups; however, no statistically significant differences were found. The highest increase in ABP was found in the Histoacryl Flexible group (217 mmHg ±53), followed by the Glubran 2 group (205 mmHg ±67). Omnex showed the lowest ABP of the TA groups (173 mmHg ±69).

### Histological evaluation

**Fig. 1:**
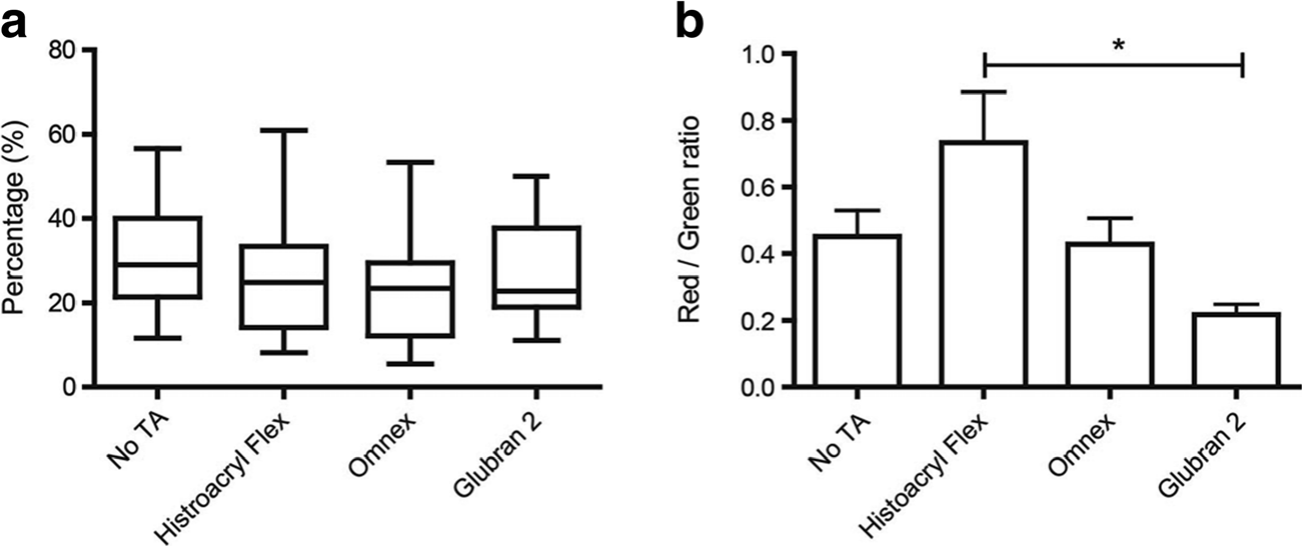
**a** Significantly more inflammation occurred in the Omnex and Glubran 2 group compared to the control group. **b** No differences were found between groups regarding fibroblast acitivity. **c** More collagen deposition was found in the Histoacryl group and the Omnex group compared to the control group. Neoangiogenesis (**d**) did not differ between the experimental groups

Histological evaluation is depicted in Fig. 1.

## Discussion

**Fig. 2:**
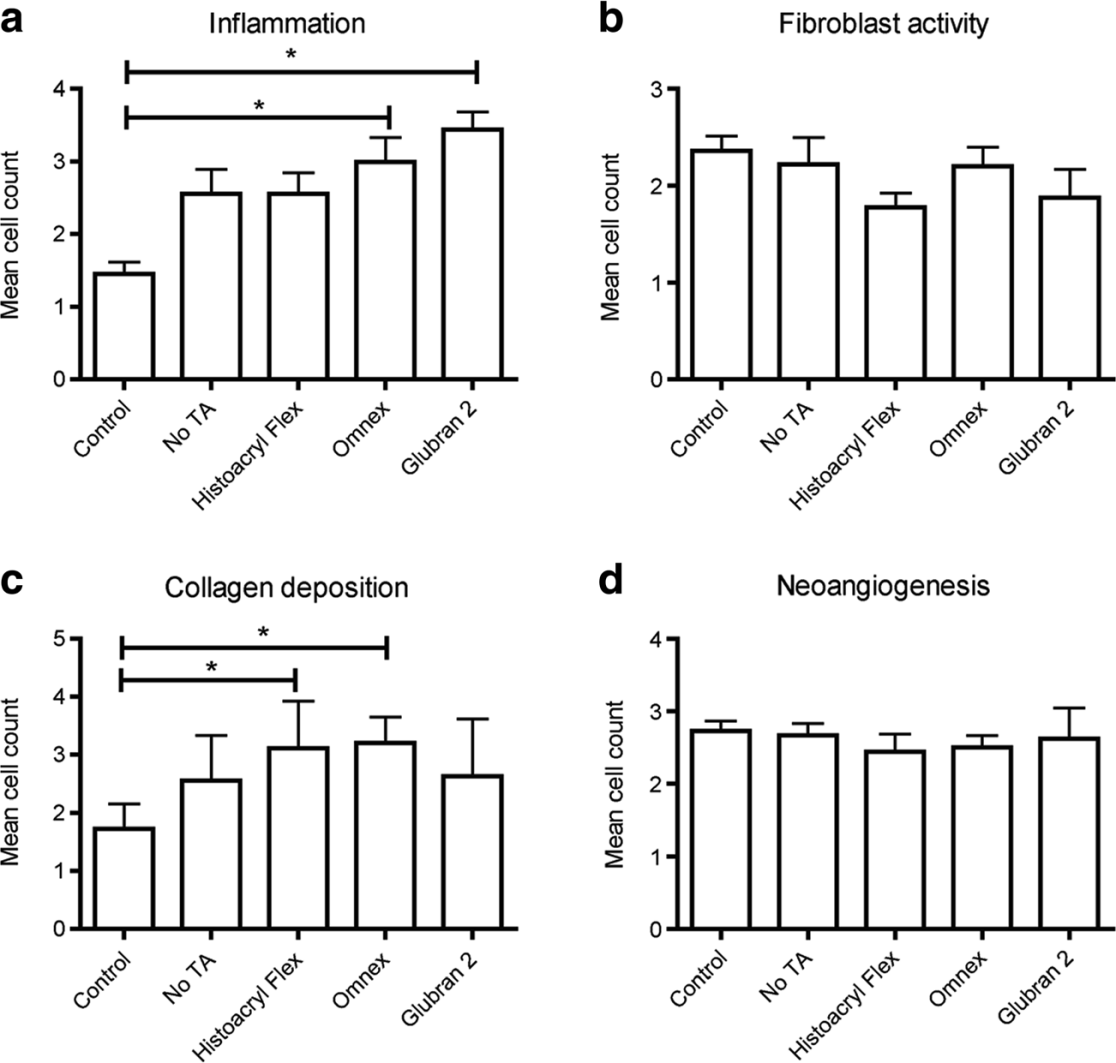
**a** No differences were found between groups in the relative collagen area (quantified as the percentage of total tissue surface). **b** Maturity of collagen was estimated by calculating the red/green ratio, which was significantly higher in the Histoacryl Flexible group compared to the Glubran 2 group, indicating more mature collagen

Overall, there were large differences between AL rates of the various groups. AL mostly presented as peri-anastomotic abscess formation. The described abscess score was used to score the severity and amount of abscesses; in this study, an abscess score of <2 was not associated with any clinical symptoms, and therefore not considered clinically relevant. Histoacryl Flexible, a combination of n-butyl-2-cyanoacrylate and a softener, showed the lowest rate of AL of the TA groups, occurring in two rats. Furthermore, the maximum abscess score in Histoacryl Flexible was lower than in the other TAs and consisted only of punctiform abscesses around the anastomosis, which did not have any clinical consequences. The histological evaluation showed that this TA resulted in the least inflammation and the highest level of collagen formation and healing of the TA groups. Overall, Histoacryl Flexible showed promising results, with an AL rate comparable to the 12-suture control group, with positive clinical outcomes and improved histological assessment. This TA seems to be a safe and effective colonic sealant (Fig. 2).

Glubran 2, based on an n-butyl-2-cyanoacrylate and methacryloxy sulfolane mixture, showed the poorest results in our study. In terms of AL, the use of this TA resulted in one case of premature death due to fecal peritonitis, as well as the highest rates of abscess formation and abscess severity. Furthermore, its use was associated with a higher incidence of mechanical ileus. Rats in this group showed the most weight loss of all study groups. Glubran 2 induced an extended inflammatory response with mild local muscle lysis as deep as the submucosal colonic layer. This finding was also reported in a previous study by Kayaoglu [12]. Omnex, a 2-octyl-cyanoacrylate/butyl-lactoyl-cyanoacrylate mixture, showed similar results to the negative control group in terms of AL rate, clinical effects, mechanical strength and histological analysis. The presence of this TA thus did not improve outcomes nor lead to any complications when used on the colon. As the follow-up time in this study was limited to 7 days, we propose a future study to focus on the long-term effects on these TAs on AL rate and tissue healing.
